# High Temperature Tribological Performance of Steel/Copper Friction Pairs Lubricated with a Modified C-WS_2_-(Fe_3_O_4_ + TiN) Nanoadditives in Non-Copper Coated Solid Wires

**DOI:** 10.3390/nano12122091

**Published:** 2022-06-17

**Authors:** Hong Li, Jing Zhu, Zisong Chen, Zhuoxin Li, Bo Meng

**Affiliations:** 1 Faculty of Materials and Manufacturing, Beijing University of Technology, Beijing 100124, China; 18810925009@163.com (J.Z.); vertism@163.com (Z.C.); zhxlee@bjut.edu.cn (Z.L.); 2 Shandong Juli Welding Co., Ltd., Dezhou 253011, China; mengbo@163.com

**Keywords:** nanoparticle, steel/copper friction pairs, tribological properties, lubrication mechanism

## Abstract

In this study, four kinds of nanoparticles, graphite, WS_2_, Fe_3_O_4_, and TiN, were used as lubricating additives for steel/copper friction pairs to solve the problem of welding contact tube wear with non-copper-coated solid wire at high temperature. The single and composite nanoparticles have excellent dispersion stability in absolute ethanol under the action of the compound surfactant NaSTA + OA + PVP (i.e., sodium stearate, oleic acid, and polyvinylpyrrolidone). The tribological test results showed that the maximum decrement, with reference to the average coefficient of friction and wear volumes, were measured with nanoparticle concentration in 1:1:1 ratio at 300 °C. Compared with dry friction, the average friction coefficient and wear volume are reduced by 74.3% and 84.8%, respectively, which may be attributed to the formation of a stable tribo-film mainly composed of C–O, Fe_2_O_3_, WO_3_, TiO_2_, TiN_x_O_y_ composite on the worn surface. Therefore, it is considered that the combined lubrication effects of the ball-bearing effect, repairing of worn surfaces, and the tribo-film resulted in the lowest friction and wear.

## 1. Introduction

Environmentally-friendly non-copper-coated solid wires are an inevitable trend in the development of robotic automatic arc welding [1]. Compared with traditional copper plating solid wires, non-copper-coated solid wires have many advantages such as less environmental pollution, beautiful shape, stable arc, less splash and smoke, good rust resistance, stable wire feeding, and energy saving, etc. [2,3,4]. However, there are problems such as serious wear of the contact tube, poor wire feedability, and poor arc stability, which reduce the precision and quality of robot automatic welding and are the bottleneck of robot welding engineering applications [5,6]. The key to solving these problems is to improve the surface coating quality of the welding wire. Nanoparticles (NPs) have small size effect, surface effect, quantum effect and macroscopic quantum tunneling effect. In theory, it is feasible to apply nanocoatings on the surface of non-copper-coated solid wires to solve the wear problem of the contact tube, but coating quality (e.g., uniform coating thickness, good bond strength of coating to substrate) depends greatly on coating techniques. Conventional coating processes (such as spraying, vapor deposition, composite plating, brush plating, etc.) cannot achieve rapid deposition of NPs and the cost is high, and some deposition processes pollute the environment.

With the combination of nanotechnology and lubricant research and development, nanomaterials used as lubricant oil additives can effectively improve the tribological properties. Currently, non-copper-coated solid wires mainly use nanomaterials as surface coating. Wesling et al. [7] studied the influence of the surface coating elements of the welding wire on the mechanical properties of the arc and weld metal. Cr coating caused arc length shortening and the tensile strength increased by 44%, and the fatigue strength of the weld metal with Cr and Ti coatings also significantly increased. Li et al. [8] found that using a mixture of nano-graphite, MoS_2_ and Fe_3_O_4_ as the surface coating of the non-copper coated solid wires can significantly reduce the wear of the contact tube at room temperature, and the mass loss rate of the contact tube is as low as 0.35%.

Temperature is an important factor that affects the performance of the surface coating of the welding wire and the wear of the contact tube [9,10]. For robotic welding using non-copper coated wire, the current continuously passes through the interface between the welding wire and the contact tube, resulting in Joule heating effect, which not only increase the local surface temperature, but also changes the viscosity and lubrication behavior of the lubricant film on the surface of the welding wire, and has a certain degree of influence on the wear of the contact tube. Take, for example, when welding at 300 A, the temperature can reach 450 °C at a distance of 2 mm from the copper-free wire and the contact tube. However, the current research on the tribological properties of carbon steel–copper alloy friction pairs is mainly in the range of room temperature to 100 °C, and there are fewer studies on the tribological behavior at higher temperatures. Furthermore, most of the new lubricants have different properties, enhanced by adding a specific proportion of special compound additives in lubricant oil at room temperature. However, there are few studies on the effect of temperature on the tribological behavior of steel–copper friction pair as the proportion of composite nano-lubricating additives changes. Dan et al. [11] prepared WS_2_/GP nanocomposites and illustrated that the friction coefficient and wear rate of WS_2_/GP at 100 °C was reduced by 70.2% and 65.8% at 0.02~0.04 wt.% compared with the base oil. The additives form a layer-by-layer mixed structure, forming a synergistic lubrication mechanism at high temperatures. An et al. [12] reported the tribological properties of ZnO doped with WS_2_ nanoparticles at room temperature and high temperature. ZnO nanoparticles have little effect on the friction coefficient of WS_2_ at 25 °C. At 400 °C, the ZnO-WS_2_ component produces a strong conversion of tribochemical reaction at high temperature and exhibits an unstable friction coefficient, while pure WS_2_ shows a lower and more stable trend.

Due to the physical and chemical properties of nanoparticles (large specific surface area, high diffusibility, easy sintering, low melting point, high hardness, etc. [13,14]), it not only forms a low-shear-stress film on the surface of the friction pair which contributes to reduce the friction coefficient, but also has a repairing effect on the worn surface [15,16]. Therefore, it has excellent anti-wear and anti-friction properties, and has great application potential as a lubricating additive in many fields. The effect of anti-wear and anti-friction performance depends on the type of additive and the number of additives. Furthermore, the optimal ratio of nanoadditives is also a key factor. A high concentration of nanoadditives can cause surface damage due to extensive wear and higher frictional heat. At present, the content of nanoadditives is generally in the range of 0.1~3% [17,18]. However, one of the defects of nanoadditives is their poor dispersibility in the lubricating medium. The key to exerting the excellent lubricating properties of NPs on the surface of the welding wire is to improve the dispersion stability in the base oil. Modification of NPs can obtain nano-lubricating oil with high dispersibility. Therefore, the particle size, proportion, and dispersibility of nanoparticles would be the key focus of further research to increase the dispersion stability of lubricating nano-additives.

Several studies have been conducted on the friction and wear of steel/copper and steel/steel friction pairs under lubrication or dry friction conditions [19,20]. Generally, steel wears relatively little due to its higher hardness during sliding. The wear of copper is relatively high, and plastic deformation is serious [21]. However, in the search for the most effective nanoadditive ratio and its tribological performance and lubrication mechanism of steel/copper under high temperature, only a few have been verified and characterized. Therefore, the study of the friction and wear of nanoparticles in steel/copper friction pair at high temperature has important theoretical significance for reducing the wear of copper contact tube and improving the welding performance of steel non-copper coated solid wires.

This work aims to investigate the high temperature tribological performance of modified C-WS_2_-(Fe_3_O_4_ + TiN) as a lubricant additive for low-carbon steel and copper alloy Cu-Cr-Zr friction pair. High temperature tribological tests are conducted under boundary lubrication to simulate the face contact between non-copper coated solid wires and contact tube. The effects of individual and hybrid nanoadditive components on the tribological properties of steel/copper friction pairs at 300 °C were studied. Finally, the lubrication mechanism of nanoparticles at the steel/copper friction interface was analyzed.

## 2. Materials and Methods

### 2.1. Materials

The C-WS_2_-(Fe_3_O_4_ + TiN) nanoadditives with an average size of 20~40 nm were synthesized by surface modification method. The final prepared C-WS_2_-(Fe_3_O_4_ + TiN) nanocomposite lubricants were added into absolute ethanol solution and stirred with the addition of surfactants dispersion to achieve stable dispersions. The surfactants are sodium stearate (NaSTA), oleic acid (OA), and polyvinylpyrrolidone (PVP). In order to fully mix the nanoparticles, surfactants, and absolute ethanol, the experiment uses a combination of ultrasonic vibration dispersion and constant temperature water bath heating for dispersion. The 1 wt.% designed nanoparticles of different components and contents and 2 wt.% surfactants were added to absolute ethanol, and ultrasonic treatment was performed for 20 min at 50 °C. Among them, the compound surfactants were prepared according to NaSTA:OA:PVP = 1:1:1, and an appropriate amount of cyclohexane was added to promote mutual dissolution of the surface modifier. The nanoadditives designed in the experiment are shown in Table 1.

### 2.2. Tribological Tests

**Figure 1:**
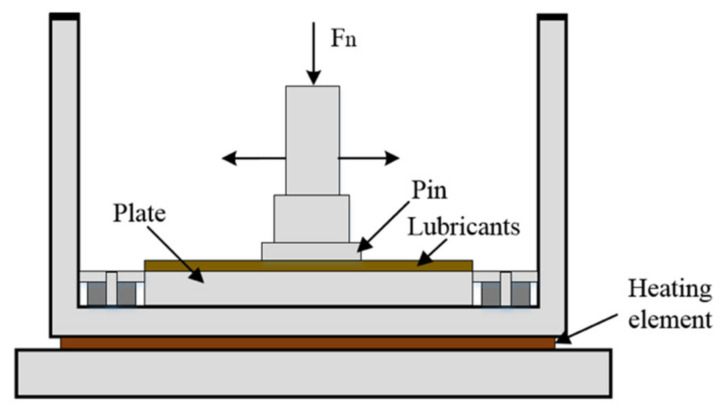
Schematic diagram of the high temperature tribological tests.

The high temperature tribological property was tested using a pin-on-plate in a reciprocation model (UMT-3, Bruker Nano Inc., Billerica, MA, USA). Schematic diagram of the high temperature tribological tests was shown in Figure 1. Sliding experiments were performed with loading force 2.5 N, displacement 10 mm, and frequency 2 Hz for 30 min at high temperature 300 °C. Each experiment was repeated two times per condition. The temperature and friction coefficient were automatically recorded by computer. The chemical composition of the friction pair material is shown in Table 2. The test steel pin was Q235 (Φ8 mm × 16 mm, surface roughness was 0.02–0.10 μm), and copper plate was Cu-Cr-Zr (40 mm × 30 mm × 6 mm, surface roughness was 0.05–0.20 μm). The friction pair samples were polished by sandpaper before tribological test, and then were ultrasonically cleaned to meet the requirements of the testing machine for the friction surface roughness and ensure the stable operation under high speed and load.

### 2.3. Characterization Methods

When the nanoadditives were fully dispersed, the absorbance of the stable supernatant of the ethanol mixture was measured after standing for 1 h, and the dispersion stability of nanoparticles in absolute ethanol was investigated. The absorbance of the supernatant was measured with an ultraviolet-visible-near-infrared spectrophotometer (UH-4150), and the scanning wavelength was 400~850 nm. Fourier infrared spectrometer (FT-IR, VERTEX 70) was used to measure the infrared spectra of different nanoparticles before and after modification, and the scanning range was 500~4000 cm^−1^. The position and intensity of the absorption peak of the molecular structure of the characterizing substance determines the chemical group of the analyte.

The worn surfaces images of copper plate were obtained by Scanning Electron Microscopy (SEM, Hitachi S4800, Fukuchiyama, Japan). The wear volumes were calculated by using a three-dimensional non-contact surface profiler (ST400, NANOVEA, Irvine, CA, USA). The three-dimensional (3D) profiles and roughness of worn surface were characterized by a laser scanning confocal microscope (CLSM, Olympus LEXT OLS4100, Tokyo, Japan). X-ray photoelectron spectroscopy (XPS, Thermo escalab 250Xi, Waltham, MA, USA) was used to analyze the structure of oxide friction film.

## 3. Results and Discussions

### 3.1. Dispersion Analysis of Nanoparticles

**Figure 2:**
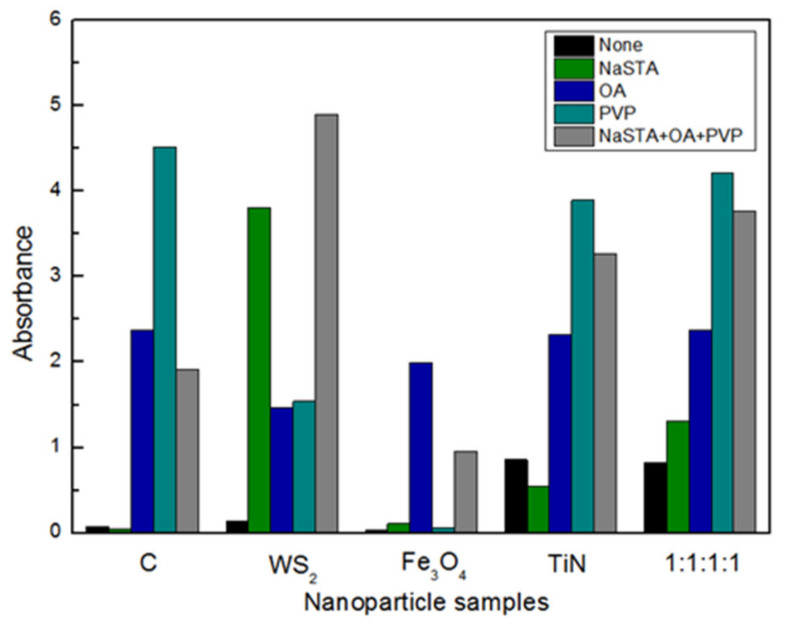
Effect of modifier types on the dispersion stability of different nanoparticles.

Different additives are more likely to agglomerate when they are compounded, which affects their performance. This effect is obvious between different elements but is not evident between the same elements. Figure 2 shows the effect of different surfactants on four kinds of nanoparticles. The larger the spectrophotometer, the better and more stable the dispersion of modified nanoparticles in absolute ethanol. Different modifiers have different effects on these four kinds of nanoparticles. PVP has a significant effect on graphite. WS_2_ is modified by NaSTA and OA, and has excellent dispersion stability. NaSTA and PVP have a poor effect on Fe_3_O_4_ modification. The overall order of dispersion effect in absolute ethanol from high to low is: PVP, OA, NaSTA. It shows that the compound surfactants have a more excellent dispersion effect on the composite nanoparticles.

**Figure 3:**
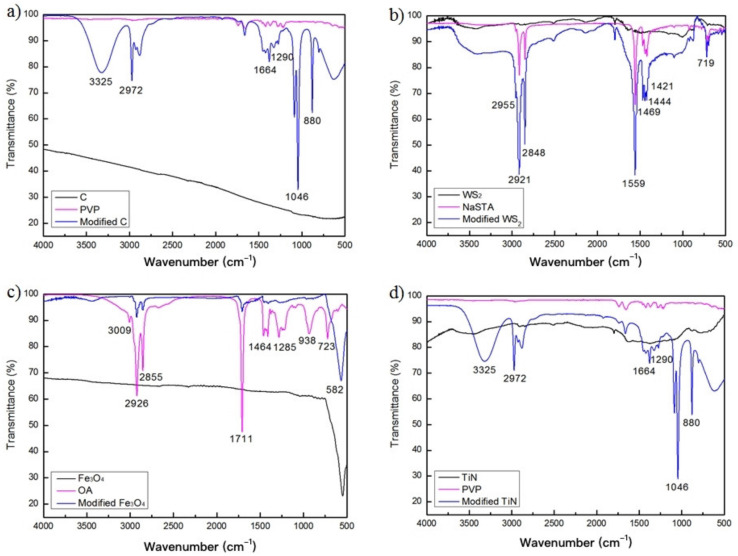
Infrared spectra of four modified nanoparticles (**a**) C-PVP, (**b**) WS_2_-NaSTA, (**c**) Fe_3_O_4_-OA, (**d**) TiN-PVP.

For the most suitable surfactants for the four kinds of nanoparticles, qualitative research before and after modification was carried out using infrared spectroscopy. Figure 3a,d shows the position around 3325 cm^−1^ is the typical absorption peak of O–H stretching vibration in water, the strong absorption broad peak at 2972 cm^−1^ is C–H stretching vibration, and the strong peak at 1450~1420 cm^−1^ is caused by methylene –CH_2_– bending vibration absorption peak. Around 1290 cm^−1^ is the characteristic peak of the amide group (–CONH–) of the PVP molecule. Comparing the two curves before and after modification of WS_2_ and Fe_3_O_4_ (Figure 3b,c), it can be seen that the peak shape of the infrared spectrum of the modified nanoparticles has changed significantly. The absorption peak at 2848 cm^−1^ indicates the presence of methylene –CH_2_–, 1469 cm^−1^ is the stretching vibration of −CH_2_−. It can be seen that the characteristic absorption peaks of long-chain functional groups similar to the surfactant appear on the four kinds of nanoparticles after modification, indicating that the surface modifier has successfully modified the nanoparticles. The main chain of the three surfactants is the hydrophobic segment of the C–C bond and the presence of non-polar methylene –CH_2_– has lipophilicity. The hydrophilic group allows it to be well adsorbed on the surface of the nanoparticles, while the lipophilic group allows the nanoparticles to be well suspended in absolute ethanol.

### 3.2. Tribological Behavior of Individual and Hybrid Nanoadditives

**Figure 4:**
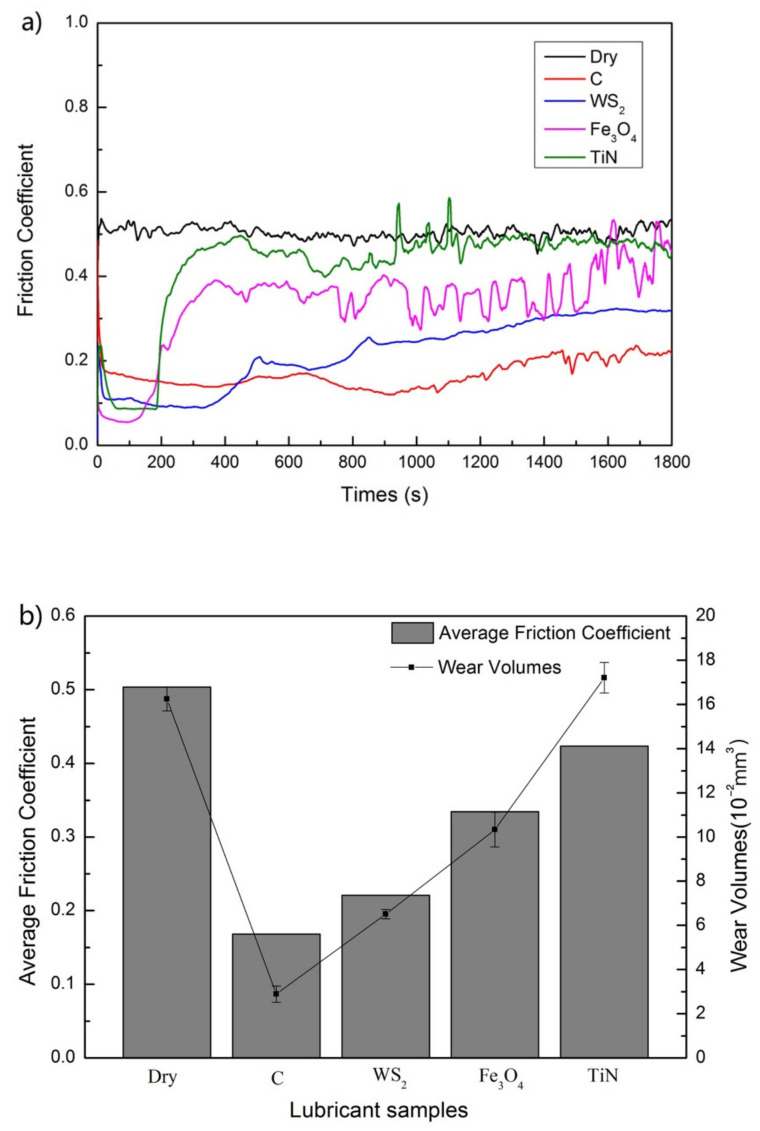
(**a**) Compare the variation of coefficient of friction with time for single nano-lubricants, (**b**) average friction coefficient and wear volumes of different single nano-lubricants.

The results show that graphite and WS_2_ have excellent anti-friction and anti-wear performance, and the wear process is stable at 300 °C, while the friction coefficient under the separate action of Fe_3_O_4_ and TiN is abnormal and the wear extent increases, see Figure 4. It shows that the anti-wear and anti-friction effect of single nano-graphite is more significant, and the friction coefficient under the action of graphite is as low as 0.1682. The anti-wear performance under nano-TiN lubrication is the worst, and the wear volume is as high as 17.2059 × 10^−2^ mm^3^. Therefore, the individual component action of nano-modified graphite and WS_2_ at 300 °C can significantly improve the anti-friction and anti-wear performance of steel/copper. In addition, for TiN lubricants, after 200 s, the friction coefficient began to increase greatly, and severe abrasive wear occurred. TiN hard particle has better thermal stability, which will generate a large number of abrasive particles and debris. When the abrasive particles and debris no longer overcome the resistance, they will be directly embedded in the friction pair matrix, making the surface wear more serious than that in dry friction condition.

**Figure 5:**
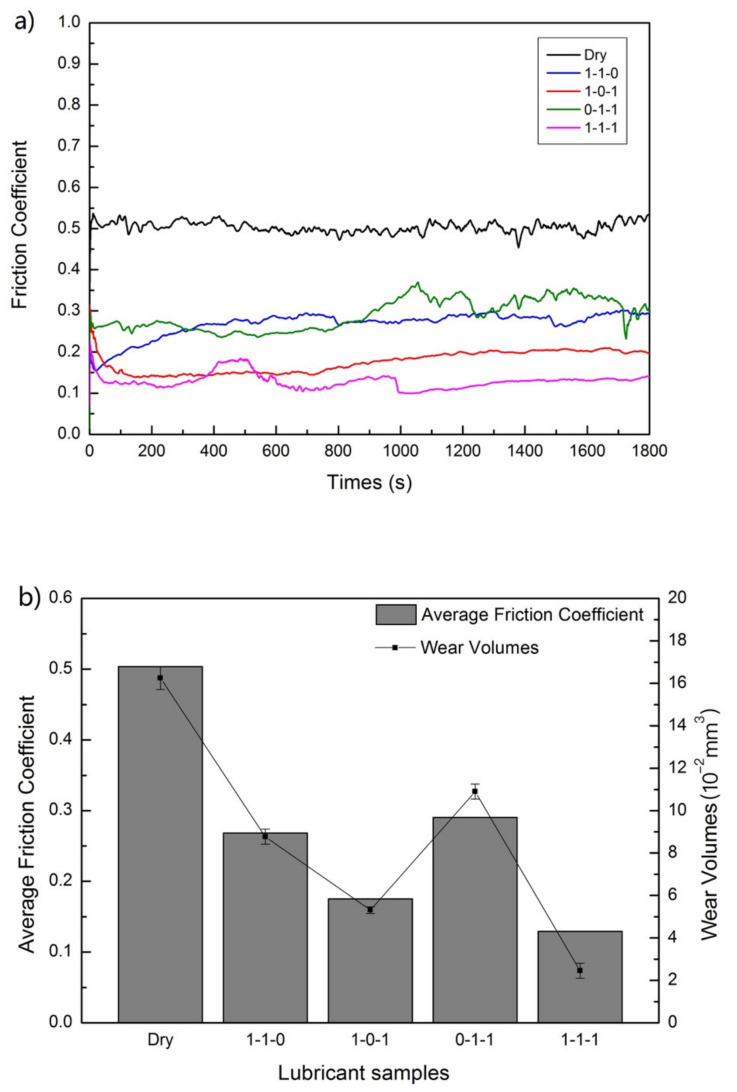
(**a**) Variation of coefficient of friction with time for nanocomposite lubricants, (**b**) average friction coefficient and wear volumes of different nanocomposite lubricants.

According to Figure 5, it shows that under the same experimental conditions, the anti-friction performance of the hybrid nanoadditives is better than that of the individual nanoadditives. Compared with dry friction, the average friction coefficient of the nanoadditive component of C:WS_2_ = 1:1, C:(Fe_3_O_4_ + TiN) = 1:1, WS_2_:(Fe_3_O_4_ + TiN) = 1:1, C:WS_2_:(Fe_3_O_4_ + TiN) = 1:1:1 is reduced by 46.7%, 65.2%, 46.3%, and 84.8%, respectively, and the wear volume is reduced by 32.6%, 67.3%, 40.1%, and 74.3%. Particularly, when the modified hybrid nanoadditives are C:WS_2_:(Fe_3_O_4_ + TiN) = 1:1:1, the tribological performance is the best, and excellent at 300 °C. This may be related to the interaction between nanoparticles and the distribution and thickness of the protective film formed on the surface of the steel/copper friction pair. The fluctuation of friction coefficient can be explained by more intense tribochemical reactions between four kinds of nanoparticles at 300 °C. Generally, compared to Figure 4 and Figure 5, hybrid C-WS_2_-(Fe_3_O_4_ + TiN) nanocomposite lubricants with the ratio of 1:1:1 work better than all individual nanoadditives.

### 3.3. Components Surface Morphology and Chemical Composition Analysis of the Copper Worn Surfaces

**Figure 6:**
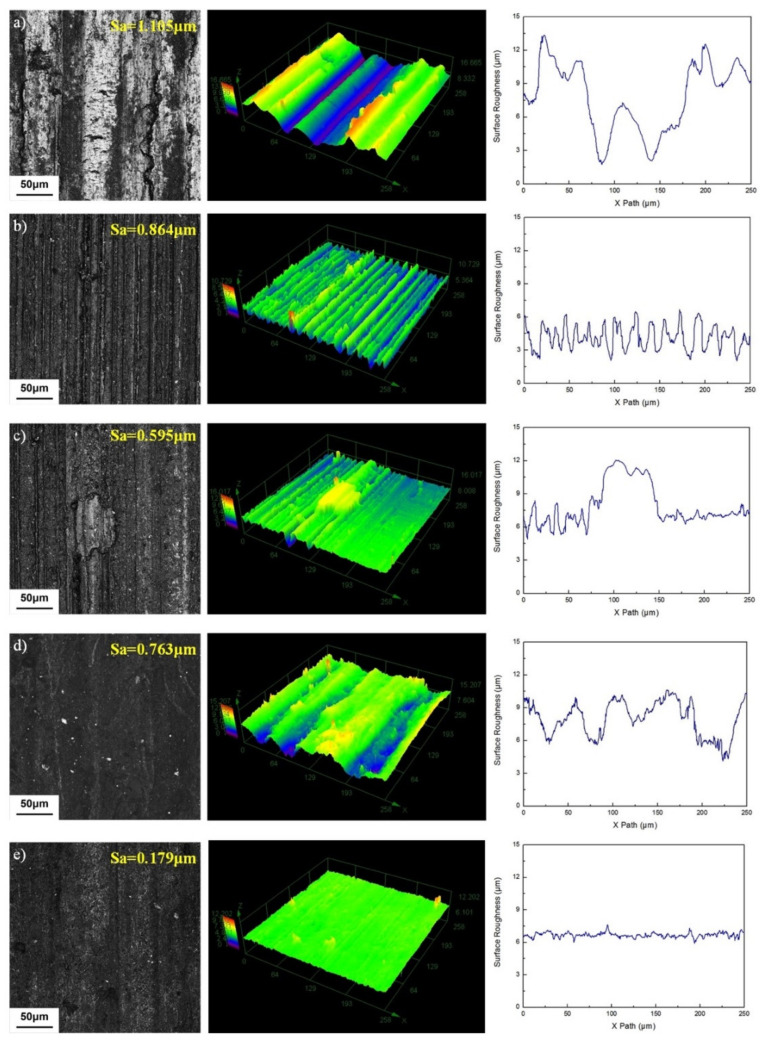
Surface topographies and roughness curves of worn surface under different lubricating conditions (**a**) Dry, (**b**) C:WS_2_:(Fe_3_O_4_ + TiN) = 1:1:0, (**c**) C:WS_2_:(Fe_3_O_4_ + TiN) = 1:0:1, (**d**) C:WS_2_:(Fe_3_O_4_ + TiN) = 0:1:1, (**e**) C:WS_2_:(Fe_3_O_4_ + TiN) = 1:1:1.

Figure 6 shows the three-dimensional surface morphology and roughness curves of Cu-Cr-Zr under different composite lubricants at 300 °C. Under the action of dry friction, the surface roughness value is the largest, which is 1.105 μm, and the surface wear of Cu-Cr-Zr is the most serious, and the measured width value of the wear scar is the largest, see Figure 6a. Compared with dry condition, the addition of hybrid nanoadditives has a significant impact on roughness of wear surface. For Figure 6b, under the action of C:WS_2_:(Fe_3_O_4_ + TiN) = 1:1:0, from the roughness curve, it can be seen that the measured width value of the wear scar is significantly reduced, but the wear scars are deeper and the grooves are more dense. Furthermore, under the action of C:(Fe_3_O_4_ + TiN) = 1:0:1, see Figure 6c, the wear surface of Cu-Cr-Zr is obviously covered with a layer of flake material and its thickness has exceeded the depth value of the wear scar, and the surface roughness is relatively large. Figure 6d shows that under the action of C:WS_2_:(Fe_3_O_4_ + TiN) = 0:1:1, the width value of the wear scar of the Cu-Cr-Zr worn surface is larger, the furrows are deeper, and the surface roughness value is larger compared with Figure 6c. When the modified hybrid nanoadditive is C:WS_2_:(Fe_3_O_4_ + TiN) = 1:1:1, as shown in Figure 6e, the surface profile is smooth, but there are still micro-furrows, and the corresponding surface roughness value is 0.179 μm. Compared with dry friction, it is reduced by 83.8%, which shows that the modified hybrid nanoadditives exhibit a good anti-friction and anti-wear effect, avoid direct metal contact, and can inhibit obvious wear on the surface of Cu-Cr-Zr. Through the cross-sectional profile curve comparison, it is shown that the surface of Cu-Cr-Zr has been improved to different degrees after adding the hybrid nanoadditives. The probable reason is that the deposition of nanoparticles produces a thin protective layer or tribofilm on the contact surfaces [13,14]. The direct contact between copper repairs the micro-damage of the worn surface during the friction process, and makes the worn surface smooth without obvious scratches, cracks, and other defects so the surface roughness and cross-sectional profile curve are smoother.

**Figure 7:**
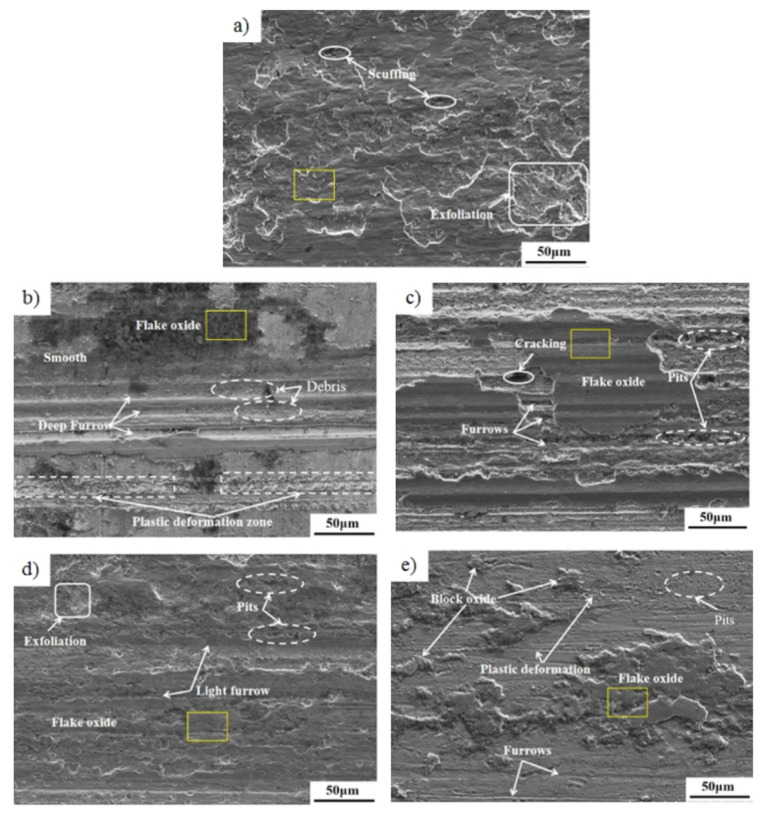
SEM morphologies of worn surfaces with different modified hybrid nanoadditives at 300 °C (**a**) Dry, (**b**) 1-1-0, (**c**) 1-0-1, (**d**) 0-1-1, (**e**) 1-1-1.

To further investigate the wear mechanism, Figure 7 shows the SEM images of the lubricant samples obtained for the Cu-Cr-Zr worn surface. The surface is found to be smoother and there is no delamination of the layers. The addition of the nanoparticles results in less wear as they provide a defensive film between the surfaces during steel and copper blocks moving. However, a further increase in nanoparticles results in a larger amount of wear volumes. Figure 7a shows that the worn surface is severely damaged and accompanied by a large amount of lamellar flaking. The flaked hard particles are embedded in the wear scar under dry friction. As shown in Figure 7b–d, under the action of different composite lubricants, there are a small amount of wear debris and massive adhesion materials on the wear surface of Cu-Cr-Zr, and there are obviously a large number of flake oxides and a small amount of block oxides, indicating the existence of friction film. When the content of nanoparticles added separately is optimal C:WS_2_:(Fe_3_O_4_ + TiN) = 1:1:1, the average friction coefficient and wear scar diameter of the friction pair can be minimized, the friction contact surface is smoothest, the shallowest grooves and scratches, and solid lubrication is significantly weakened, inducing decreased wear (Figure 7e). It mainly shows that when the ratio of the four kinds of nanoparticles in the friction process at 300 °C is an average ratio, a tribochemical reaction is generated to form a more uniformly distributed friction film, which reduces the direct contact of the friction pair.

**Figure 8:**
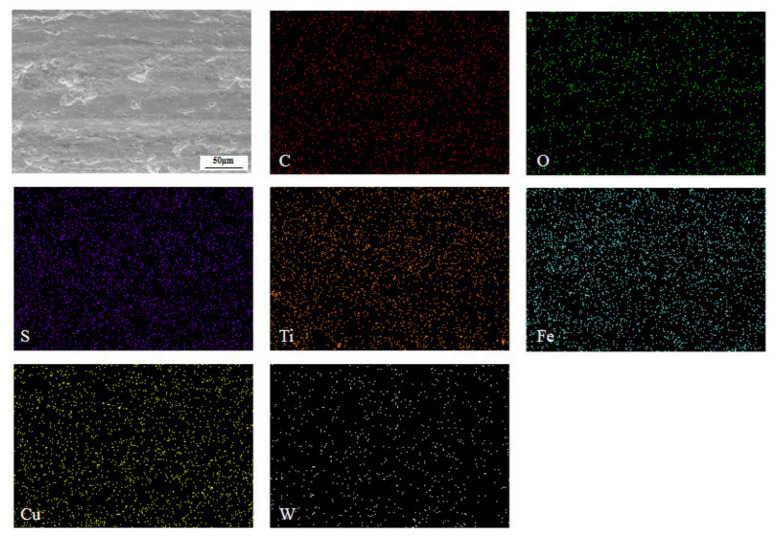
Elemental mapping analysis of the tribofilm identified in Figure 7e.

**Figure 9:**
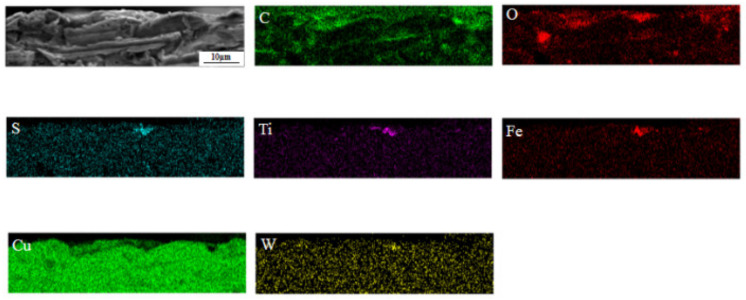
EDS surface scanning of C:WS_2_:(Fe_3_O_4_ + TiN) = 1:1:1 on the Cu-Cr-Zr wear section.

It can be seen from the surface element distribution (Figure 8) that C:WS_2_:(Fe_3_O_4_ + TiN) = 1:1:1 modified hybrid nanoadditives have a uniform distribution of C, Fe, Ti, and W elements on the wear surface, indicating that the graphite, WS_2_, Fe_3_O_4_, and TiN nanoparticles added at 300 °C reacted with the matrix material of the friction pair to form a uniform repairing film containing four elements. The friction film separates the friction pair, avoids direct contact between steel and copper, effectively inhibits the further occurrence of surface wear, and explains the reason for different distribution, size, and roughness of the friction film in the scanning electron microscope image. In addition, the reduction of Cu element indicates that the nanoparticles did not react with the material of friction pair when the friction surface moved relative to each other. As shown in Figure 9, the anti-friction and anti-wear effect is due to the small size of the nanoparticles, which can play the role of micro-bearing. The nanoparticles and oxides are embedded in the pits and damaged parts of the friction interface during the friction process, which play a role in repairing the rough surface, making the worn surface smooth and reducing the surface roughness.

**Figure 10:**
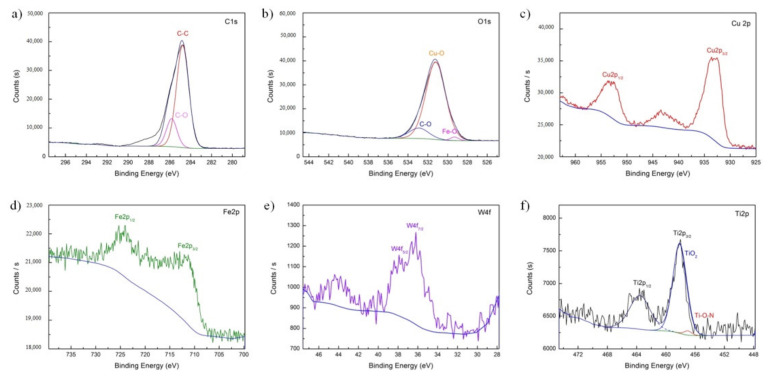
XPS spectrum of the worn surface lubricated with C:WS_2_:(Fe_3_O_4_ + TiN) = 1:1:1. (**a**) C1s, (**b**) O1s, (**c**) Cu2p, (**d**) Fe2p, (**e**) W4f, (**f**) Ti2p.

The XPS fine spectrum analysis results of the wear surface elements under the action of C:WS_2_:(Fe_3_O_4_ + TiN) = 1:1:1 modified hybrid nanoadditive are shown in Figure 10. The full spectrum mainly detected elements such as Cu, Fe, O, Ti, and C, which were consistent with the results of EDS. O1s mainly produces copper oxide and carbon oxide during the friction process. It can be seen from Figure 10b,d that the Fe2p peak with a binding energy of 710.9 eV and the O1s peak with a binding energy of 530.2 eV are attributed to Fe_2_O_3_, and the Fe2p peak with a binding energy of 708.1 eV and O1s with a binding energy of 529.3 eV The peak of the spectrum is attributed to FeO, indicating that a chemical reaction film containing Fe_2_O_3_ and FeO is formed on the surface of the friction pair through tribochemical action. Analyzing Ti2p, two peaks of 456.79 eV and 458.85 eV are fitted at the Ti2p_3/2_ peak (Figure 10f), which are the corresponding peaks of Ti-containing oxides, representing TiN_x_O_y_ and TiO_2_, respectively.

By analyzing the above-mentioned elements, it can be seen that a substance contains multiple oxides such as Cu, C, Fe, W, and Ti, etc. It shows that the four kinds of nanoparticles added chemically react during the friction process of the steel/copper friction pair, forming an extremely thin repairing film composed of CO, Fe_2_O_3_, WO_3_, TiO_2_, and TiN_x_O_y_ on the worn surface, which plays a role in repairing the worn surface of Cu-Cr-Zr.

### 3.4. Lubrication Mechanisms

**Figure 11:**
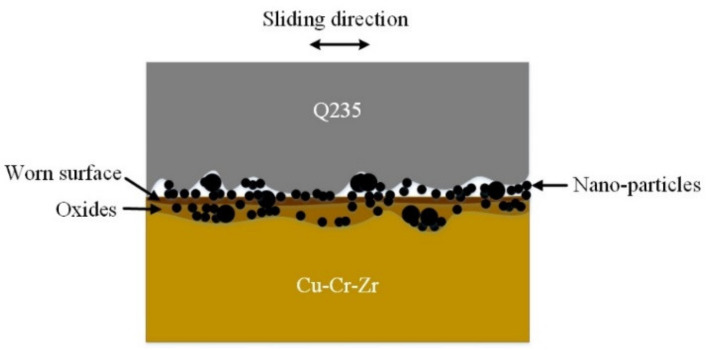
Model of lubrication mechanism of steel–copper friction interface with nanoadditives as lubricants.

Figure 11 shows the lubrication mechanism of the steel/copper friction interface. During the friction process at 300 °C, the nanoparticles and their decomposition products aggregate, adsorb, and react on the surface, forming a friction film, which plays a role of lubrication and protection and is the mechanism of decomposition and film formation. Nanoparticles are extremely small in size, and they are embedded in the pits of the friction interface during the friction process to repair the rough surface and make the worn surface smooth. When the low carbon steel and Cu-Cr-Zr slide relatively, the nanoparticles have a ball-bearing effect. Hence, the modified hybrid nanoadditives generate a tribochemical reaction at the friction interface at high temperature to form a repairing film, which shows good anti-wear and anti-friction and repairing properties.

## 4. Conclusions

The deaggregation and prolonged dispersion of four kinds of nanoparticles are modified by surface modification with NaSTA, OA, and PVP in absolute ethanol. The modified nanoparticles exhibit very stable dispersion at a high absorbance value, compared with the untreated nanoparticles. The dispersion stability of the compound surfactant NaSTA + OA + PVP is obviously better than that of the single surfactant. Stable dispersion of nanoparticles in a lubricating medium can significantly improve its anti-friction and anti-wear performance.

Compared with dry friction, individual and hybrid nanoadditives can reduce the amount of wear and friction coefficient, improving the anti-friction and anti-wear properties of steel/copper friction pair. Particularly, hybrid nanoadditives C:WS_2_:(Fe_3_O_4_ + TiN) = 1:1:1 is superior to others individual and hybrid nanoadditives in improving the anti-wear and anti-friction performance Cu-Cr-Zr. Its average friction coefficient and wear volume are reduced by 84.8% and 74.3%, respectively, the worn surface is the smoothest, and the surface roughness value is the lowest.

The lubrication mechanism for steel/copper friction pairs with modified C-WS_2_-(Fe_3_O_4_ + TiN) hybrid lubricants at high temperature may be identified as that, when low-carbon steel and Cu-Cr-Zr are relatively sliding, the four kinds of nanoparticles at the friction interface interact with tribochemical reactions at 300 °C to form a friction film composed of CO, Fe_2_O_3_, WO_3_, TiO_2_, TiN_x_O_y_, etc. It shows excellent anti-friction and anti-wear performance and repairing effect. Different nanoparticles effectively play the role of synergistic lubrication of ball-bearing lubrication, repairing worn surfaces and film formation by tribochemical reactions. The results are promising for reducing the wear of copper contact tube and improving welding performance of steel non-copper coated solid wires.

## Figures and Tables

**Table 1:** Chemical compositions of employed lubricants.

Lubrication Type	Composition wt.%		
	C	WS_2_	Fe_3_O_4_ + TiN
1	Dry condition		
2	1	0	0
3	0	1	0
4	0	0	1
5	0.5	0.5	0
6	0.5	0	0.5
7	0	0.5	0.5
8	0.333	0.333	0.333

**Table 2:** Chemical compositions of the pin and plate materials.

Materials	Chemical Composition (wt.%)							
**Pin—Q235**	**C**	**Mn**	**Si**	**S**	**P**	**Fe**		
	≤0.17	≤1.4	≤0.35	≤0.035	≤0.035	Bal.		
Plate—Cu-Cr-Zr	Cr	Zr	Fe	Al	Mg	Si	P	Cu
	0.1- 0.8	0.3- 0.6	0.5	0.1- 0.25	0.1- 0.25	0.5	0.1	Bal.

## Data Availability

The datasets supporting the conclusions of this article are included within the article.
